# Reliability and Responsiveness of Cardiopulmonary Exercise Testing in Fatigued Persons with Multiple Sclerosis and Low to Mild Disability

**DOI:** 10.1371/journal.pone.0122260

**Published:** 2015-03-19

**Authors:** Martin Heine, Lizanne Eva van den Akker, Olaf Verschuren, Anne Visser-Meily, Gert Kwakkel, TREFAMS-ACE Study Group

**Affiliations:** 1 Brain Center Rudolf Magnus and Center of Excellence for Rehabilitation Medicine, University Medical Center Utrecht and Rehabilitation Center De Hoogstraat, Rembrandtkade 10, 3583 TM, Utrecht, The Netherlands; 2 Department of Rehabilitation Medicine & EMGO Institute for Health and Care Research, VU University Medical Center, Amsterdam, The Netherlands; 3 Department of Rehabilitation Medicine, MOVE Research Institute Amsterdam, VU University Medical Center, Amsterdam, The Netherlands; 4 Department of Neurorehabilitation, Centre of Rehabilitation and Rheumatology READE, Amsterdam, The Netherlands; University of Texas at Dallas, UNITED STATES

## Abstract

**Background:**

Peak oxygen uptake (VO_2_peak) via cardiopulmonary exercise testing is considered the gold standard for testing aerobic capacity in healthy participants and people with various medical conditions. The reliability and responsiveness of cardiopulmonary exercise testing outcomes in persons with MS (PwMS) have not been extensively studied.

**Objective:**

(1) to investigate the reliability of cardiopulmonary exercise parameters in PwMS; (2) to determine the responsiveness, in terms of the smallest detectable change (SDC), for each parameter.

**Design:**

Two repeated measurements of cardiopulmonary exercise outcomes were obtained, with a median time interval of 16 days.

**Methods:**

Thirty-two PwMS suffering from subjective fatigue performed cardiopulmonary exercise tests on a cycle ergometer, to voluntary exhaustion. We calculated the reliability, in terms of the intra-class correlation coefficient (ICC [2,k]; absolute agreement), and the measurement error, in terms of standard error of measurement (SEM) and SDC at individual (SDC_individual_) and group level (SDC_group_).

**Results:**

The ICC for VO_2_peak was 0.951, with an SEM of 0.131 L∙min^−1^ and an SDC_individual_ of 0.364 L∙min^−1^. When corrected for bodyweight, the ICC of VO_2_peak was 0.933, with an SEM of 1.7 mL∙kg^−1^∙min^−1^ and in an SDC_individual_ of 4.6 mL∙kg^−1^∙min^−1^.

**Limitations:**

Generalization of our study results is restricted to fatigued PwMS with a low to mild level of disability.

**Conclusions:**

At individual level, cardiopulmonary exercise testing can be used reliably to assess physical fitness in terms of VO_2_peak, but less so to determine significant changes. At group level, VO_2_peak can be reliably used to determine physical fitness status and establish change over time.

## Introduction

Multiple sclerosis (MS) is a chronic demyelinating inflammatory disorder of the central nervous system (CNS) with unknown etiology.[1] Progressive demyelination within the CNS results in a range of motor, sensory, cerebellar and cognitive dysfunctions, many of which may, directly or indirectly, limit physical fitness. The resulting impairments and limitations in daily activities may subsequently result in considerable physical deconditioning and even in comorbidities that reduce life expectancy.[2–4] Maintaining or improving physical fitness in persons with MS (PwMS) is paramount. In the past two decades, several intervention studies have investigated the feasibility and potential benefits of various types of exercise training in PwMS.[5–8] These studies have showed that training regimes like endurance, resistance or combined training can be used safely by PwMS. In addition, these training regimes may prevent deconditioning or improve physical fitness in these patients.[5–8] Measuring physical fitness is important in the context of clinical practice to evaluate people’s health status, and in the context of research to evaluate the effects of exercise regimes or other interventions.[7] Cardiopulmonary exercise testing (CPET) using maximal cycling ergometry is considered the ‘gold standard’ for the assessment of exercise tolerance in both healthy people and people with various medical conditions.[9] CPET is preferably accompanied by measurements of ventilation and gas exchange (i.e. VO_2_peak) for direct assessment of aerobic function.[10] If a measure is to be used in clinical practice, it should be both content-valid and reliable. In this case, consecutive measurements from a given subject who has not changed, should be the same under several conditions (test-retest).[11]

Recently, we showed that CPET is a valid measure of physical fitness in PwMS with a low to mild level of disability (Expanded Disability Status Scale; EDSS ≤ 4.0).[12] In addition, we showed that the Oxygen Uptake Efficiency Slope (OUES), as a potential alternative to VO_2_peak in PwMS who are unable to attain maximal exercise intensities, is highly predictive of VO_2_peak.[13] Even though the validity and the responsiveness of CPET in PwMS have not been studied extensively until recent[8, 12, 14], the CPET has been used as an outcome in clinical trials quite often already.[8, 15–20] For instance, in a study by Petajan et al., a 22% improvement in VO_2_peak was found following a 15 week aerobic training program.[20] In contrast, Mostert and Kesseling were unable to replicate these results, in terms of VO_2_peak, in a shorter but more intensive training program.[19] However, little is known about the extent to which maximal exercise performance in PwMS is hampered by day-to-day variation. In other words, how confident are we that the reported 22% change in VO_2_peak is not due to measurement error? To our knowledge, one study has suggested that CPET can be used reliably in PwMS under stringent laboratory conditions.[14] This study, by Langeskov-Christensen and colleagues reported that a change of ~10% is required to consider this change significant. However, MS is an unstable medical condition with symptom experience changes even within days, yet little is known on the reliability and responsiveness of CPET in a less stringent laboratory setting which may, for instance, substantially affect our confidence in the 22% change in VO_2_peak as reported by Petajan et al.[20] This question is important in the light of clinical research and practice, as the responsiveness of a test may provide valuable information on the required number of participants (i.e. statistical power) in clinical trials using CPET, on the ability of the test to determine individual changes in VO_2_peak in clinical practice, and to determine if the change in VO_2_peak following a clinical trial can be considered beyond measurement error. Our research question was therefore: what is the reliability and responsiveness of CPET, and in particular VO_2_peak, as assessed by two consecutive CPETs when applied to PwMS?

The objective of the present study was twofold: (1) to investigate the reliability of CPET, and in particular VO_2_peak, in PwMS; (2) to determine the responsiveness, in terms of the smallest detectable change.

## Participants and Methods

### Participant selection

A joint convenience sample was composed from patients treated at two study centers (VU Medical Center, Amsterdam, the Netherlands and St. Antonius Medical Center, Nieuwegein, the Netherlands). The sample consisted of 32 participants of the TREFAMS-ACE study (Treating Fatigue in Multiple Sclerosis: Aerobic Therapy, Cognitive Behavioral Therapy, Energy Conservation Management; ISRCTN69520623 and ISRCTN58583714) with definite MS.[21] No a priori power analysis was conducted. The sample size of 32 was reached by requesting participation in this reliability study of all TREFAMS-ACE participants who attended the first or second follow-up measurement between august 2012 and October 2013. The TREFAMS-ACE study and the present reliability study were approved by the Medical Ethics Board of the VU University Medical Center Amsterdam. Each potential participant received both written and oral information about the TREFAMS-ACE trial before providing written informed consent. In addition, participants provided supplementary consent for participation in the present reliability study, which involved an additional exercise test. To be included in the TREFAMS-ACE study, participants had to experience substantial fatigue (≥ 35 on the fatigue subscale of the Checklist on Individual Strength[22]), had to be ambulant (≤ 6.0 on the Expanded Disability Status Scale [EDSS][23]) and had to have no clinical depression (≤ 11 on the Depression subscale of the Hospital Anxiety and Depression Scale[24]). Potential participants were excluded if they had participated in a professionally supervised therapy program to alleviate fatigue in the three months before inclusion. Potential participants were also excluded if they had comorbidities precluding maximal exercise participation, or MS relapse confirmed by a neurologist in the three months prior to study participation. Details of the TREFAMS-ACE study have been described elsewhere.[21]

Prior to the cardiopulmonary exercise test, self-reported baseline characteristics and disease specifics were recorded: sex, age, height, weight, body mass index (BMI), time since diagnosis and type of MS (classified as relapsing-remitting, primary progressive, secondary progressive or not specified). Neurological disability was determined by a certified physician using the EDSS.[23]

### Cardiopulmonary exercise testing (CPET)

Two identical incremental exercise tests were performed on a programmable, electromagnetically braked cycle ergometer (Kettler X7, Heinz Kettler, Germany) to determine maximal exercise capacity. Within each study center, one assessor conducted the test and re-test. The two exercise tests were conducted during the follow-up phase of the TREFAMS-ACE study, but at least 10 weeks after the intervention phase. A period of one to three weeks was scheduled between the two consecutive tests, depending on the participants’ flexibility in making an appointment. Participants were asked not to change their exercise habits between the two tests. During the intervention phase of the TREFAMS-ACE study, participants had already performed at least one exercise test, and were thus familiar with the test protocol. To avoid bias, the two assessors in each center and the participants were not informed of the results of the first test prior to the second test.

Handlebars and saddle of the cycle ergometer were adjusted to match each participant’s anthropometrics. Following a 3-minute rest phase, participants started cycling at 25 Watt, with power output increasing by 10 Watt (women) or 15 Watt (men) each minute. This protocol was based on the rationale that small increments prolong exercise duration and enable participants with peripheral limitations to sustain longer exercise duration. Preferably, the exercise was terminated by voluntary exhaustion following 8–12 minutes of exercise.[25] During the exercise testing, participants were asked to maintain a cadence of 60–80 rotations per minute (rpm). Participants were verbally encouraged, especially beyond a respiratory exchange ratio > 1.00. The exercise test was terminated by voluntary exhaustion, by a cadence < 45 rpm or for safety reasons, compliant with the American College of Sports Medicine’s guidelines for clinical exercise testing.[26]

Each participant’s gas exchange was measured using a portable mixing-chamber monitoring system (Cortex MetaMax 3B; Cortex Medical; Germany). The Cortex Metamax is a valid and reliable system for measuring ventilation parameters during exercise.[27, 28] Volume, pressure, and gas analyzers were calibrated automatically by the system prior to each test using a 3L syringe, atmospheric pressure at the time (mmHg), and both ambient and reference gases (4.98% CO_2_, 17.05% O_2_), respectively. Raw data was averaged over 10-second intervals for analysis. Peak exercise values were defined as the highest recorded 10-second average. The following parameters derived from the CPET were used in the present study: peak oxygen consumption (VO_2_peak), maximal work output (Wmax), maximal ventilation (VEmax), maximal respiratory exchange ratio (RER), perceived exertion immediately following voluntary exhaustion, measured by the 20-point BORG scale[29], and maximal heart rate. In the current literature VO_2_peak is often, but not always, expressed relative to bodyweight. Hence, the present study used both the corrected and uncorrected VO_2_peak. In addition, we included the Oxygen Uptake Efficiency Slope (OUES). The OUES may be a valuable submaximal measure of physical fitness in those PwMS for whom VO_2_peak is a measure of performance, rather than aerobic capacity.[13] The OUES was determined by measuring the slope of VO_2_ (mL∙kg^−1^∙min^−1^) and log_10_ VE (L∙min^−1^) until voluntary exhaustion. The OUES has been claimed to be independent of exercise duration, exercise protocol and assessor.[30, 31] It has also been shown that the OUES is highly predictive of VO_2_peak in PwMS.[13]

### Statistics

From a psychometric point of view, reliability can be defined as the proportion of total variance in the measurements which indicates a ‘true’ difference between patients.[32] In this concept, each observation is assumed to be composed of two components: a true score and an error associated with the association.[11] Based on this definition, the proportion of between-patient variance compared to the total variance was calculated by the intra-class correlation coefficients (ICC [2, k]) model (i.e., a two-way random model with absolute agreement). An ICC greater than 0.80 reflects excellent reliability, whereas ICCs from 0.70 to 0.79 reflect good reliability.[33] The recommended minimum for the lower limit of the 95% confidence interval (CI) is 0.85.[34] Measurement error was defined as the systematic and random error in a patient’s score that is not attributed to true changes in CPET performance. The standard error of measurement (SEM_agreement_) was used for the measurement error, to determine the precision of the total score of both tests. The SEM describes the error in interpreting an individual’s test score. It allows the ‘true’ test performance to be estimated using a reliability coefficient and is computed by multiplying the standard deviation of the total score by the square root of 1 minus its reliability coefficient (SEM = SD_pooled_ x √1—ICC).[35] The smallest detectable change (SDC_individual_) in each aerobic capacity outcome was computed as 1.96 x √2 x SEM to obtain a 95% CI.[32, 35] In addition we considered it valuable, especially for clinical research, to determine the smallest detectable difference at group level (SDC_group_) rather than at individual level. The SDC_group_ was calculated as SDC_individual_ / √*n*. The Bland–Altman procedure was used to check for heteroscedasticity of the test and retest for each outcome measure.[36] All analyses were conducted using the SPSS statistical package (version 19.0).

## Results

A total of 13 men and 19 women (Table 1) completed both tests without complications. The median disability level on the EDSS scale was 2.5 (Interquartile range (IQR) 2.0–3.0) Most participants had relapsing-remitting MS (N = 27). The second test was conducted after a median interval of 16 days (IQR 14–19 days) following the first.

**Table 1:** Participant characteristics.

Participant characteristics	N = 32	SD (if mean)
		IQR (if median)
Sex (N) men women	1319	
Type of MS (N)Relapsing-Remitting Primary ProgressiveSecondary ProgressiveNot specified	27122	
Mean age, years	50.7	8.1
Mean height, cm	176	11
Mean weight, kg	79	15
Mean BMI, kg/m^2^	25.6	3.7
Median time since diagnosis, years	9	4–13
Median EDSS score	2.5	2.0–3.0
Median days between tests	16	14–19

BMI, Body Mass Index; EDSS, Expanded Disability Status Scale; IQR, Interquartile range; N, number of participants; SD, standard deviation.

### Reliability and responsiveness

**Fig 1:**
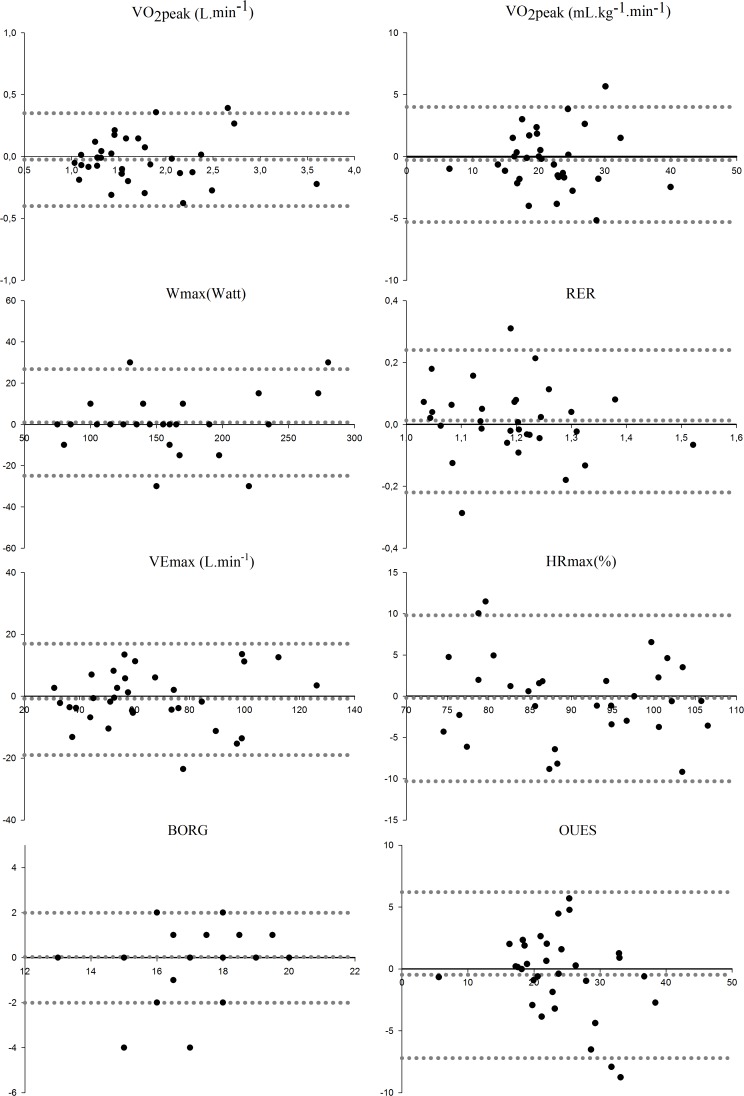
Bland-Altman plots of CPET outcomes in PwMS. BORG, rating of perceived exertion following voluntary exhaustion; HRmax, maximal heart rate expressed as percentage of the age-predicted maximal heart rate (%); OUES, oxygen uptake efficiency slope; RER, respiratory exchange ratio; VE, peak ventilation (L∙min^−1^); VO_2_peak, peak oxygen uptake (L∙min^−1^and mL∙kg^−1^∙min^−1^); Wmax, maximal power output (Watt).

The reliability and responsiveness of the CPET outcomes are presented in Table 2. The reliability of both the %HRmax and the BORG score was based on 31 instead of 32 participants due to one missing value, as the heart rate monitor lost signal during the exercise test for one participant, and the BORG score was not acquired within one minute post voluntary exhaustion for another participant. The ICC of VO_2_peak was 0.951, with an SEM of 0.131 L∙min^−1^and an SDC_individual_ of 0.364 L∙min^−1^. After correcting for body weight, the ICC for VO_2_peak was 0.933, with an SEM of 1.7 mL∙kg^−1^∙min^−1^ and an SDC_individual_ of 4.6 mL∙kg^−1^∙min^−1^. The ICC for maximal power was 0.971, with an SEM of 9.1W and an SDC of 25.2W. As regards peak ventilation, the ICC was found to be 0.936, with an SEM of 6.4 L/min and an SDC_individual_ of 17.7 L/min. As can be seen from the Bland-Altman plots (Fig. 1) there were no signs of heteroscedasticity, except for the OUES. Potential outliers were included in the analysis.

**Table 2:** Reliability and responsiveness of CPET in 32 PwMS.

	Test 1	Test 2							
Outcome	Mean (SD_1_)	Mean (SD_2_)	MD (SD_pooled_)	ICC	95%CI of ICC	SEM	SDC_individual_	SDC_group_	LOA
VO_2_peak (L∙min^−1^)	1.745 (0.593)	1.720 (0.593)	−0.03 (0.19)	0.951	0.902 to 0.976	0.131	0.364	0.06	−0.40 to 0.35
VO_2_peak (mL∙kg^−1^∙min^−1^)	21.7 (6.4)	21.5 (6.4)	−0.29 (2.36)	0.933	0.868 to 0.967	1.7	4.6	0.82	−5.0 to 4.3
Wmax (Watt)	157.8 (51.6)	158.8 (53.4)	0.94 (12.92)	0.971	0.941 to 0.985	9.1	25.2	4.46	−24.9 to 26.8
RER	1.19 (0.13)	1.20 (0.12)	0.01 (0.12)	0.565	0.273 to 0.761	0.08	0.23	0.04	−0.22 to 0.24
VE (L/min)	66.0 (24.7)	65.1 (25.4)	−0.81 (9.04)	0.936	0.874 to 0.968	6.4	17.7	3.13	−19 to 17
%HRmax (n = 31)	90.6 (10.8)	90.3 (9.8)	−0.22 (5.03)	0.886	0.777 to 0.943	3.5	9.8	1.73	−10.3 to 9.8
BORG (n = 31)	17.4 (1.8)	17.6 (2.0)	0.03 (1.45)	0.706	0.466 to 0.849	1.1	2.9	0.52	−2.9 to 2.9
OUES	24.1 (7.5)	23.6 (6.4)	−0.48 (3.34)	0.887	0.782 to 0.943	2.4	6.6	1.16	−7.2 to 6.2

BORG, rating of perceived exertion following voluntary exhaustion; HRmax, maximal heart rate expressed as percentage of the age-predicted maximal heart rate (%); ICC, Intra-class Correlation Coefficient including 95% Confidence Interval (95%CI); LOA, Limits Of Agreement; OUES, oxygen uptake efficiency slope; RER, respiratory exchange ratio; SD, Standard Deviation; SDC, Smallest Detectable Change; SEM, Standard Error of Measurement; VE, peak ventilation (L∙min^−1^); VO_2_peak, peak oxygen uptake (L∙min^−1^and mL∙kg^−1^∙min^−1^); Wmax, maximal power output (Watt).\

## Discussion

The purpose of the present study was twofold: (1) to investigate the reliability of cardiopulmonary exercise parameters in PwMS; (2) to determine the responsiveness, in terms of the smallest detectable change, of each parameter. The aerobic capacity, in terms of VO_2_peak, was low (21.6 ± 6.4 mL∙kg^−1^∙min^−1^) when compared to reference values for sedentary healthy people (39.0 ± 6.8 mL∙kg^−1^∙min^−1^ for sedentary men; 30.0 ± 5.4 mL∙kg^−1^∙min^−1^ for sedentary women).[37] The present study showed that the reliability of VO_2_peak, Wmax and VE could be considered excellent. However, we found that some individuals may need large changes in aerobic capacity (>21%) for them to be considered real changes beyond the 95%CI intervals of measurement error.

### Reliability and responsiveness

The excellent reliability in two consecutive tests of VO_2_peak, as shown by the high ICCs, is in line with ICCs previously reported for CPET.[38, 39] Langeskov-Christensen and colleagues found a Pearson correlation coefficient of 0.98, which is of the same magnitude as the ICC found in the present study, for two consecutive CPETs in a sample of PwMS with similar disability (mean EDSS = 2.6).[14] In contrast to the excellent test-retest reliability, the smallest detectable change for an individual PwMS was relatively large which questions the responsiveness of this measure. A change of at least 0.367 L∙min^−1^ or 4.6 mL∙kg^−1^∙min^−1^ (~21%) was required for it to be considered a statistically significant improvement or deterioration. This may indicate that the incremental exercise test at individual level may not be suitable for monitoring change in aerobic capacity in PwMS. However, the small SDC_group_ found in the present study suggests that, at group level, small changes in the aerobic capacity of PwMS can be identified. The SDC_individual_ in the present study (~21%) was considerably larger than the required change found in the study by Langeskov-Christensen et al. (~10%).[14] One possible explanation is that this may be related to the more stringent standardization in the study by Langeskov-Christensen et al. (in terms of food intake, time between tests and exercise behavior).[14] A second possible explanation is that the present study used two different exercise labs, which may have introduced some additional variance. Thirdly, the present study recruited PwMS who experienced excessive fatigue. Since there was no restriction on various factors that may have influenced fatigue, perceived fatigue may have been different on each test occasion, which may explain some of the variance between the tests. However, on the contrary, the participants of the present study were familiar with the testing protocol due to the fact that this reliability study was done during the follow-up phase of an intervention study which also included CPET. Hence, this should have reduced the likelihood of a familiarization effect during the second test and reduced the between-test variance in comparison to for example Langeskov-Christensen et al.[14]

Recently, it was shown that VO_2_peak is a valid outcome for physical fitness in fatigued PwMS with a low to mild level of disability.[12] In the present study, the mean RER and %HRmax confirm that, in PwMS and low to mild disability the CPET can be considered a valid measure of aerobic capacity.[12, 14] However, the outcome of CPET in PwMS with a moderate level of disability may be more closely related to function than to physical fitness.[12] We have suggested the oxygen uptake efficiency slope as an alternative measure of physical fitness, which can also be used in case of submaximal exercise duration.[13] An ICC of 0.93 has been previously reported for the OUES in healthy people.[40] However, the present study suggests a relatively high day-to-day variation in oxygen uptake efficiency in PwMS. A change of ~27% is needed, based on the results of the present study, for it to be considered a significant change. Alternatively, the OUES may complement the assessment of VO_2_peak, as it may capture some of the physiological mechanisms (e.g. respiratory muscle work) that can explain the variation in VO_2_peak between two tests.

### Limitations

Some limitations to this study need to be addressed, which may affect the interpretation of our results and can provide clues for ways to reduce measurement error and improve reliability. First, two different exercise labs were involved, with a different assessor, use of equipment and recruitment area. Research on the interrater reliability and interlab reliability of CPET in PwMS will provide additional insights into modifiable factors to reduce measurement error. Second, some of the day-to-day variation between the tests may be related to uncontrolled sources of variance, like food and beverage intake, but also to daily differences in subjective symptoms like fatigue. Third, participants of the present study had a low to mild (EDSS < 4.0) level of disability, and experienced excessive fatigue, which restricts the generalization of our study results.

### Conclusions

At individual level, CPET can be used reliably to assess physical fitness status, but less so to determine significant changes, in terms of aerobic capacity unless the test circumstances are rigorously (e.g. timing, temperature, food and beverage intake etc.) controlled between tests. This may also include subjective symptoms like fatigue. At group level, CPET can be reliably used to determine physical fitness status and establish change. Further research is warranted on the cardiovascular working mechanisms of exercise therapy in MS, the trainability of PwMS, and clinical relevance of changes in aerobic capacity in PwMS.

## Supporting Information

S1 Dataset Data underlying the presented results. (XLS)

S1 Appendix The TREFAMS-ACE study group. (DOCX)
