# Supplementary material for: Reliability and Responsiveness of Cardiopulmonary Exercise Testing in Fatigued Persons with Multiple Sclerosis and Low to Mild Disability
Source: PLoS One. 2015 Mar 19;10(3):e0122260. doi: 10.1371/journal.pone.0122260 (PMC4366200; doi:10.1371/journal.pone.0122260)
Supplement: S1 Appendix — (DOCX) [file pone.0122260.s002.docx]

**TREFAMS-ACE Study Group**

V de Groot and H Beckerman (program coordination), A Malekzadeh, LE van den Akker, M Looijmans (until September 2013), SA Sanches (until February 2012), J Dekker, EH Collette, BW van Oosten, CE Teunissen, MA Blankenstein, ICJM Eijssen, M Rietberg. VU University Medical Center, Amsterdam;

M Heine, O Verschuren, G Kwakkel, JMA Visser-Meily, IGL van de Port (until February 2012), E Lindeman (until September 2012), Center of Excellence for Rehabilitation Medicine, University Medical Centre Utrecht and Rehabilitation Centre, De Hoogstraat, Utrecht;

LJM Blikman, J van Meeteren, JBJ Bussmann, HJ Stam, RQ Hintzen. Erasmus MC, University Medical Center, Rotterdam;

HGA Hacking, E Hoogervorst, STFM Frequin. St Antonius Hospital, Nieuwegein;

JH Knoop, BA de Jong (until January 2014), G Bleijenberg (until April 2012). University Medical Center St Radboud, Nijmegen;

FAJ de Laat, Rehabilitation Center Leijpark, Tilburg;

MC Verhulsdonck, Rehabilitation Center, Sint Maartenskliniek, Nijmegen

EThL van Munster, Jeroen Bosch Hospital, Den Bosch;

CJ Oosterwijk, GJ Aarts (until March 2013). Dutch patient organisation, Multiple Sclerosis Vereniging Nederland (MSVN), The Hague.
